# Health inequities lead to early death in many persons with disabilities

**Published:** 2023-01

**2 December 2022 - Geneva -** A new report by the World Health Organization shows evidence of a higher risk of premature death and illness among many persons with disabilities compared to others in the society. The Global report on health equity for persons with disabilities published today shows that because of the systemic and persistent health inequities, many persons with disabilities face the risk of dying much earlier - even up to 20 years earlier - than persons without disabilities. They have an increased risk of developing chronic conditions, with up to double the risk of asthma, depression, diabetes, obesity, oral diseases, and stroke. Many of the differences in health outcomes cannot be explained by the underlying health condition or impairment, but by avoidable, unfair and unjust factors.

Launched ahead of the International Day of Persons with Disabilities, the report shows the number of people with significant disabilities worldwide has risen to 1.3 billion (or 1 in 6 people). This number reinforces the importance of achieving full and effective participation of persons with disabilities in all aspects of society and embedding the principles of inclusion, accessibility and non-discrimination in the health sector.

**Unfair factors: a key cause of disparities in health**

The report stresses the need for urgent action to address the vast inequities in health caused by unjust and unfair factors within health systems. These factors - which account for many of the differences in health outcomes between persons with and without disabilities - could take the form of: I) negative attitudes of healthcare providers; II) health information in formats that cannot be understood; or III) difficulties accessing a health centre due to the physical environment, lack of transport or financial barriers. “Health systems should be alleviating the challenges that people with disabilities face, not adding to them,” said WHO Director-General, Dr Tedros Adhanom Ghebreyesus. “This report shines a light on the inequities that people with disabilities face in trying to access the care they need. WHO is committed to supporting countries with the guidance and tools they need to ensure all people with disabilities have access to quality health services.” With an estimated 80% of persons with disabilities living in low- and middle-income countries where health services are limited, addressing health inequities could be challenging. Yet even with limited resources, much can be achieved.

**Opportunities for a disability-inclusive health sector**

Recognizing that everyone has the same right to the highest attainable standard of health, the report provides important economic analysis of adopting a disability-inclusive approach. It shows investing in a disability-inclusive health sector is cost-effective. WHO calculates that governments could expect a return of about US$ 10 for every US$ 1 invested on disability-inclusive noncommunicable disease prevention and care. In addition, family planning and vaccination are cost–effective when implemented in a disability-inclusive manner.

**Targeted and comprehensive actions across the health sector**

The report outlines 40 actions across the health sector for governments to take, drawing on the latest evidence from academic studies as well as consultations with countries and civil society, including organizations representing persons with disabilities. These actions vary by resource level and range from addressing physical infrastructure to training of health and care workers. Ensuring health equity for persons with disabilities will also have wider benefits and can advance global health priorities in 3 ways: I) health equity for all is critical towards achieving universal health coverage; II) inclusive public health interventions that are administered equitably across different sectors can contribute to healthier populations; and III) advancing health equity for persons with disabilities is a central component in all efforts to protect everyone in health emergencies.

Available from: https://www.who.int/news/item/02-12-2022-health-inequities-lead-to-early-death-in-many-persons-with-disabilities

